# T-bet-dependent ILC1- and NK cell-derived IFN-γ mediates cDC1-dependent host resistance against *Toxoplasma gondii*

**DOI:** 10.1371/journal.ppat.1008299

**Published:** 2021-01-19

**Authors:** Américo H. López-Yglesias, Elise Burger, Ellie Camanzo, Andrew T. Martin, Alessandra M. Araujo, Samantha F. Kwok, Felix Yarovinsky

**Affiliations:** Center for Vaccine Biology and Immunology, University of Rochester, Rochester, New York, United States of America; University of New Mexico, UNITED STATES

## Abstract

Host resistance against intracellular pathogens requires a rapid IFN-γ mediated immune response. We reveal that T-bet-dependent production of IFN-γ is essential for the maintenance of inflammatory DCs at the site of infection with a common protozoan parasite, *Toxoplasma gondii*. A detailed analysis of the cellular sources for T-bet-dependent IFN-γ identified that ILC1s and to a lesser degree NK, but not T_H_1 cells, were involved in the regulation of inflammatory DCs via IFN-γ. Mechanistically, we established that T-bet dependent innate IFN-γ is critical for the induction of IRF8, an essential transcription factor for cDC1s. Failure to upregulate IRF8 in DCs resulted in acute susceptibility to *T*. *gondii* infection. Our data identifies that T-bet dependent production of IFN-γ by ILC1 and NK cells is indispensable for host resistance against intracellular infection via maintaining IRF8+ inflammatory DCs at the site of infection.

## Author summary

Mounting a robust type I innate immune response is essential for resistance against numerous intracellular pathogens. The type I immune response is characterized by the production of IFN-γ, a central cytokine required for multiple non-redundant effector functions against bacterial, viral, and parasitic pathogens. Previous work has shown that group 1 innate lymphoid cells (ILC1s) together with NK and CD4+ T cells play an indispensable IFN-γ-mediated protective role against *Toxoplasma gondii* infection; yet, the pathway of how IFN-γ produced by innate immune cells defends against *T*. *gondii* remains unknown. In this work, we identified that early T-bet-dependent production of IFN-γ by ILC1 and NK cells is essential for maintaining dendritic cells (DCs) during infection. Mechanistically, we reveal that T-bet controlled innate IFN-γ is indispensable for inducing the transcription factor IRF8 that is critical for sustaining inflammatory DCs. Finally, we demonstrate that IRF8+ DCs are critical for parasite elimination.

## Introduction

Host defense against intracellular pathogens requires a quick and effective type I immune response. A coordinated response of innate myeloid and lymphoid cells is critical for both rapid pathogen restriction and activation of the adaptive immune response. The transcription factor T-bet, encoded by *Tbx21*, has been shown to play a critical role for the effector function of innate and adaptive lymphocytes in response to intracellular pathogens via regulation of IFN-γ production [1–8]. The cytokine IFN-γ is indispensable for host defense as it is essential for the induction of anti-microbial IFN-γ-inducible genes, which results in intracellular microbial clearance.

The obligate intracellular protozoan parasite *Toxoplasma gondii* is a potent inducer of IFN-γ and has been exploited to characterize the host’s innate and adaptive type I immune responses against intracellular pathogens [9]. Immunity against *T*. *gondii* requires a type I CD4+ T helper cell (T_H_1)-derived IFN-γ response, and in the absence of either CD4+ T cells or IFN-γ, the host rapidly succumbs to infection [10–12]. Therefore, it was anticipated that the rapid susceptibility observed in *T*. *gondii* infected T-bet-deficient (*Tbx21*^-/-^) mice was due to the absence of CD4+ T cell-derived IFN-γ. However, our group and others have recently observed that *Tbx21*^-/-^ mice maintained IFN-γ producing CD4+ T cells during parasite infection, while remaining highly susceptible to infection [4,5]. These data suggest an innate T-bet-dependent mechanism that is critical for a protective type I immune response against *T*. *gondii* infection.

Natural killer (NK) cells and group 1 innate lymphoid cells (ILC1s) are critical sources of innate IFN-γ during intracellular infection [13,14]. It has been established that the transcription factors, Eomesodermin (Eomes) and T-bet play a role in NK cell development [15,16]. However, while Eomes is indispensable for NK cell maturation and effector function, T-bet plays a more limited role in the development, migration, and cytokine production of NK cells [4,16–18]. During *T*. *gondii* infection, NK cell-derived IFN-γ stimulates the effector function of inflammatory myeloid cells, and in the absence of NK cells the host immunity to *T*. *gondii* is compromised [13,19].

Similar to NK cells and T_H_1s, tissue resident ILC1s have been identified as a critical source of IFN-γ necessary for pathogen restriction [14,20–22]. Unlike NK cells, the maturation and cytokine production of ILC1s is T-bet-dependent and Eomes-independent [14]. Tissue resident ILC1s rapidly respond to type I conventional DC (cDC1)-derived IL-12 in an antigen independent manner, leading to IFN-γ production [22,23]. In addition to providing an early source of IFN-γ, ILC1s can also augment the recruitment of innate inflammatory myeloid cells during *T*. *gondii* infection [14], demonstrating that crosstalk between innate myeloid cells and ILC1s is a critical component of an effective innate type I immune response against intracellular pathogens.

Because multiple cellular sources of IFN-γ play a complex and non-redundant role for T-bet-dependent host defense against the common protozoan parasite *T*. *gondii*, we sought to determine the role T-bet plays in coordinating innate myeloid cells and lymphocytes to work in concert with one another for host resistance. Our experiments revealed that in the absence of T-bet, inflammatory DCs were significantly compromised at the site of infection. We identified that parasite-mediated T-bet-dependent ILC1-derived IFN-γ is crucial for maintaining inflammatory DCs during infection. In addition, NK cell-derived IFN-γ played a similar, although less profound, role in the regulation of DCs during *T*. *gondii* infection. Importantly, the absence of DCs could be rescued by exogenous administration of IFN-γ during *T*. *gondii* infection, indicating a critical role for early T-bet-dependent IFN-γ in regulating inflammatory DCs. Mechanistically, we uncovered that innate IFN-γ was required for induction of the transcription factor interferon regulatory factor-8 (IRF8) in cDC1s, which are required for immunity against *T*. *gondii*. Our results establish that during *T*. *gondii* infection, T-bet-dependent IFN-γ is indispensable for regulating inflammatory IRF8+ cDC1s, leading to parasite clearance and host survival.

## Results

### T-bet is critical to maintain inflammatory DCs during *T*. *gondii* infection

**Fig 1:**
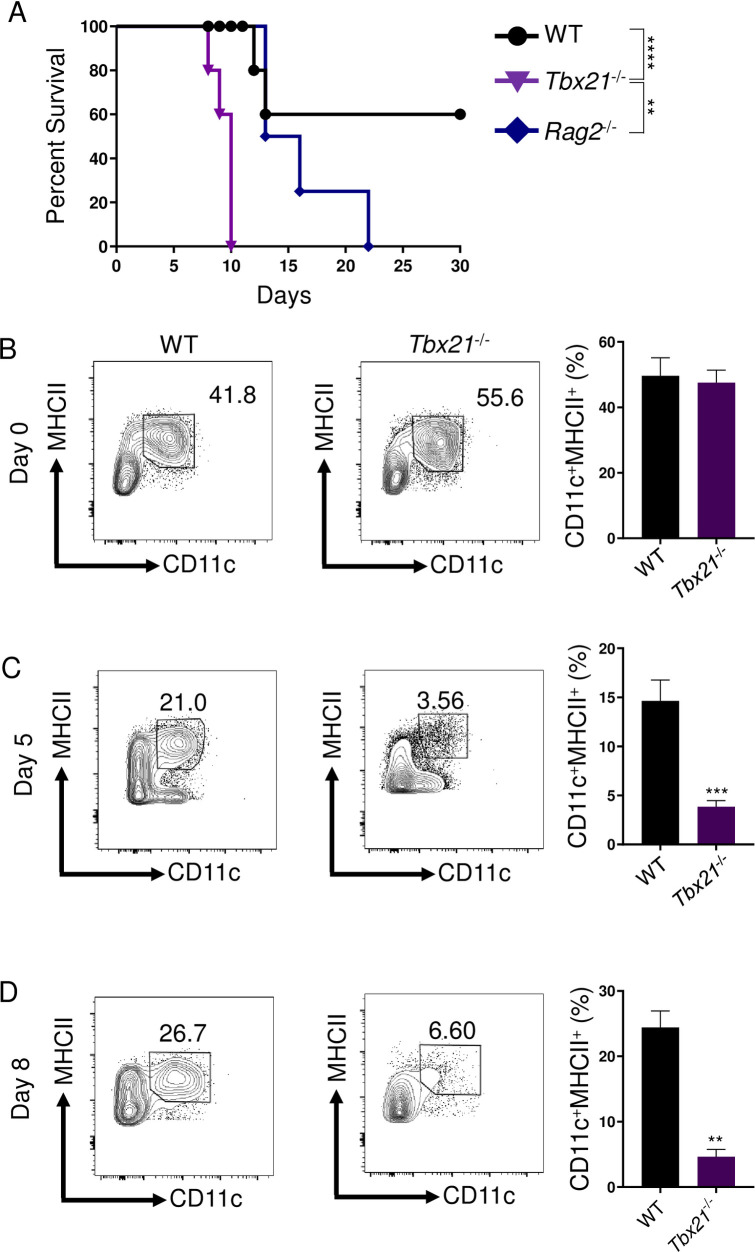
T-bet is critical for inflammatory DCs and host resistance during acute *T*. *gondii* infection. (A) Survival of WT (●), *Tbx21*^-/-^ (▼), and *Rag2*^-/-^ (♦) mice infected i.p. with 20 cysts of the ME49 strain of *T*. *gondii*. WT and *Tbx21*^-/-^ mice were i.p. infected and PECs were harvested on days 0, 5, and 8 (B, C, D) and Lin^-^CD11c^+^MHCII^+^ DCs were analyzed by flow cytometry. Average frequencies of Lin^-^CD11c^+^MHCII^+^ DCs (B, C, D) in the PECs were analyzed on days 0, 5, and 8 following infection. Results are representative of three-independent experiments involving at least 3 mice per group. Statistical analyses were done using Log-rank (Mantel Cox) test or unpaired t-test analysis of individual groups, **P<0.01, ***P<0.001, ****P<0.0001. Error bars, standard error mean.

To establish T-bet’s protective role in host defense against intracellular pathogens, we implemented the well-established intraperitoneal (i.p.) model of infection with *T*. *gondii* that triggers and depends on a robust CD4+ T_H_1-derived IFN-γ response [10,24]. Considering the importance of T-bet in the regulation of T_H_1 cells [7], we anticipated that T-bet-deficient mice would demonstrate enhanced susceptibility to *T*. *gondii* similar to T-cell deficient mice due to the lack of T-bet-mediated T_H_1-derived IFN-γ production. Strikingly, mice lacking T-bet were more susceptible to *T*. *gondii* compared to *Rag2*^-/-^ animals and were unable to survive past day 10 of the acute stage of infection (Fig 1A). These data suggested that the transcription factor T-bet is involved in innate host defense and is essential for host survival through the acute stage of parasitic infection independently of the regulation of T_H_1 immunity to the parasite.

It has been well-established that *T*. *gondii*-mediated type I immunity requires DCs for host defense [25–29]. Work from our lab and others have demonstrated that DC-deficiency results in acute host susceptibility to *T*. *gondii* [26,30]. Therefore, we examined if the absence of T-bet compromised inflammatory DC-mediated immunity. We observed that in naïve mice, lacking T-bet had no discernable effect on the presence of DCs in the peritoneal cavity (Fig 1B), defined as lineage-negative (CD3^-^CD19^-^NK1.1^-^; Lin^-^) CD11c^+^MHCII^+^ cells (S6A Fig). In striking contrast, *Tbx21*^-/-^ mice had a significantly reduced frequency of inflammatory DCs compared to both WT and *Rag2*^-/-^ animals when infected with *T*. *gondii* (Figs 1C, 1D and S1). To determine if *T*. *gondii*-mediated DCs required intrinsic T-bet expression, we employed a mouse model of DC-restricted T-bet deficiency using the CD11c-Cre system (CD11c-Cre x *Tbx21*^flox/flox^ mice, DC-*Tbx21*^-/-^). Similar to WT controls, DC-*Tbx21*^-/-^ retained Lin^-^CD11c^+^MHCII^+^ DCs during parasite infection (S2A Fig). A detailed analysis of inflammatory DCs present at the site of infection revealed that as early as day 3 post-infection, there was a noticeable reduction in DCs analyzed in *Tbx21*^-/-^ mice when compared to WT mice (S1A Fig). Furthermore, by day 5 post-infection, inflammatory DCs were practically undetectable in the absence of T-bet, a trend that was also observed by day 8 following infection (Fig 1C and 1D). Overall, our experiments established a critical innate function of T-bet for regulating the presence of inflammatory DCs during acute parasitic infection.

### Early T-bet-dependent IFN-γ is critical for inflammatory DCs via regulation of IRF8

**Fig 2:**
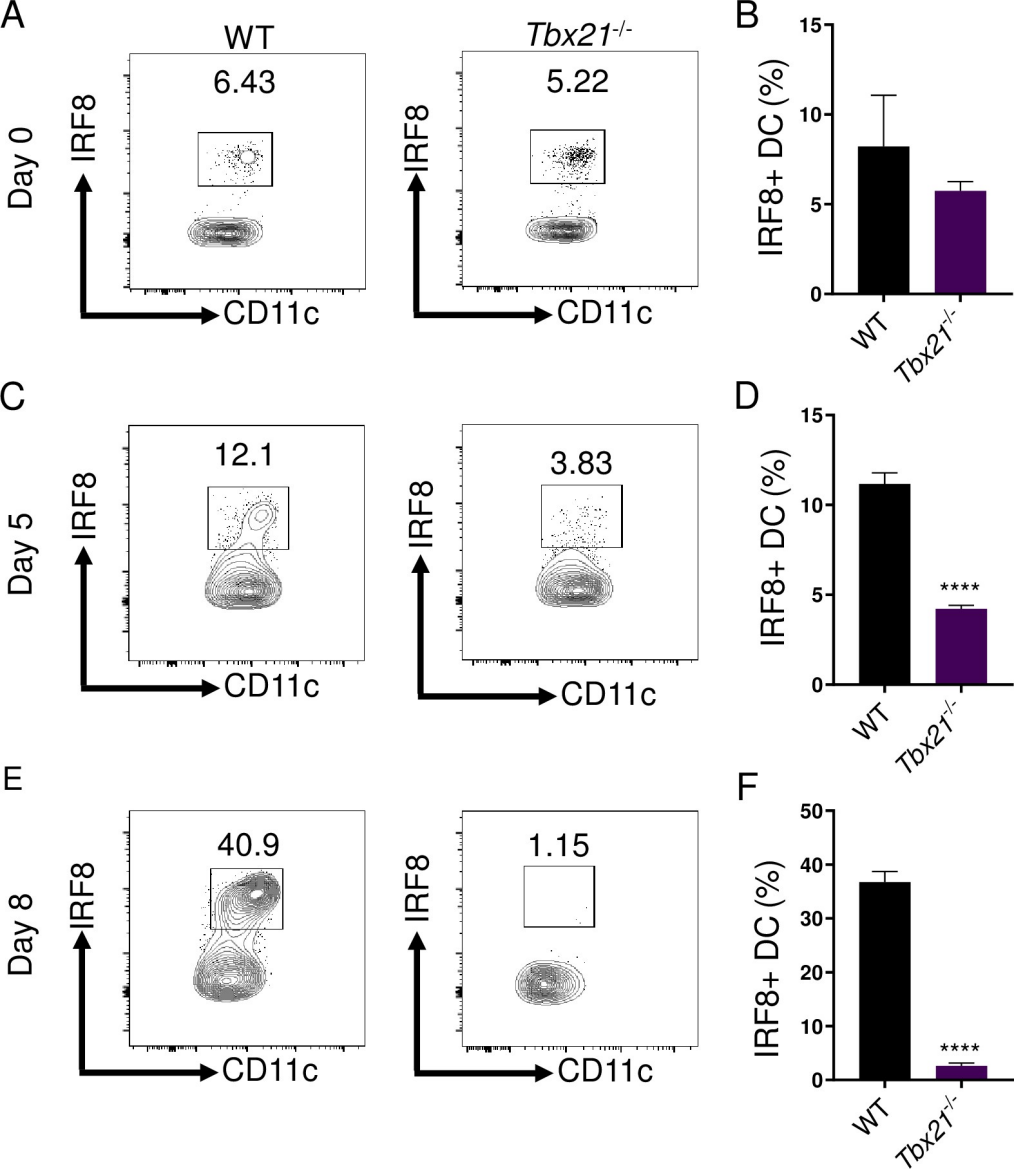
T-bet is essential for sustaining IRF8+ DCs. (**A-F**) WT and *Tbx21*^-/-^ mice were infected i.p. with 20 cysts of *T*. *gondii*. (A, C, E) Representative contour plots and (B, D, F) average frequencies of Lin^-^CD11c^+^MHCII^+^IRF8^+^ DCs in the PECs as analyzed on days 0, 5, and 8 of infection. Results are representative of three-independent experiments involving at least 3 mice per group. Statistical analyses were done using unpaired t-test analysis of individual groups, ****P<0.0001. Error bars, standard error mean.

The IFN-γ inducible transcription factor IRF8, originally described as interferon consensus sequence-binding protein (ICSBP), is known to play a key role in the development and survival of cDC1s, also known as CD8^+^BATF3^+^ DCs [31–35]. Therefore, we investigated if T-bet regulates the presence of inflammatory DCs via induction of IRF8. We observed that in naïve mice, T-bet was dispensable for the presence of a small population of peritoneal IRF8^+^ DCs, similar to WT and *Rag2*^-/-^ animals (Figs 2A, 2B and S1D). Strikingly, *T*. *gondii* infection resulted in the rapid accumulation of inflammatory IRF8^+^ DCs in WT mice and this population was virtually absent in *Tbx21*^-/-^ mice by day 8 post-infection (Figs 2C–2F and S1E). Additionally, the absence of IRF8^+^ DCs in *Tbx21*^-/-^ resulted in an overall reduction of IL-12 production during *T*. *gondii* infection (S2C Fig). These data suggest that early IFN-γ missing in *Tbx21*^-/-^ mice results in cDC1 deficiency.

**Fig 3:**
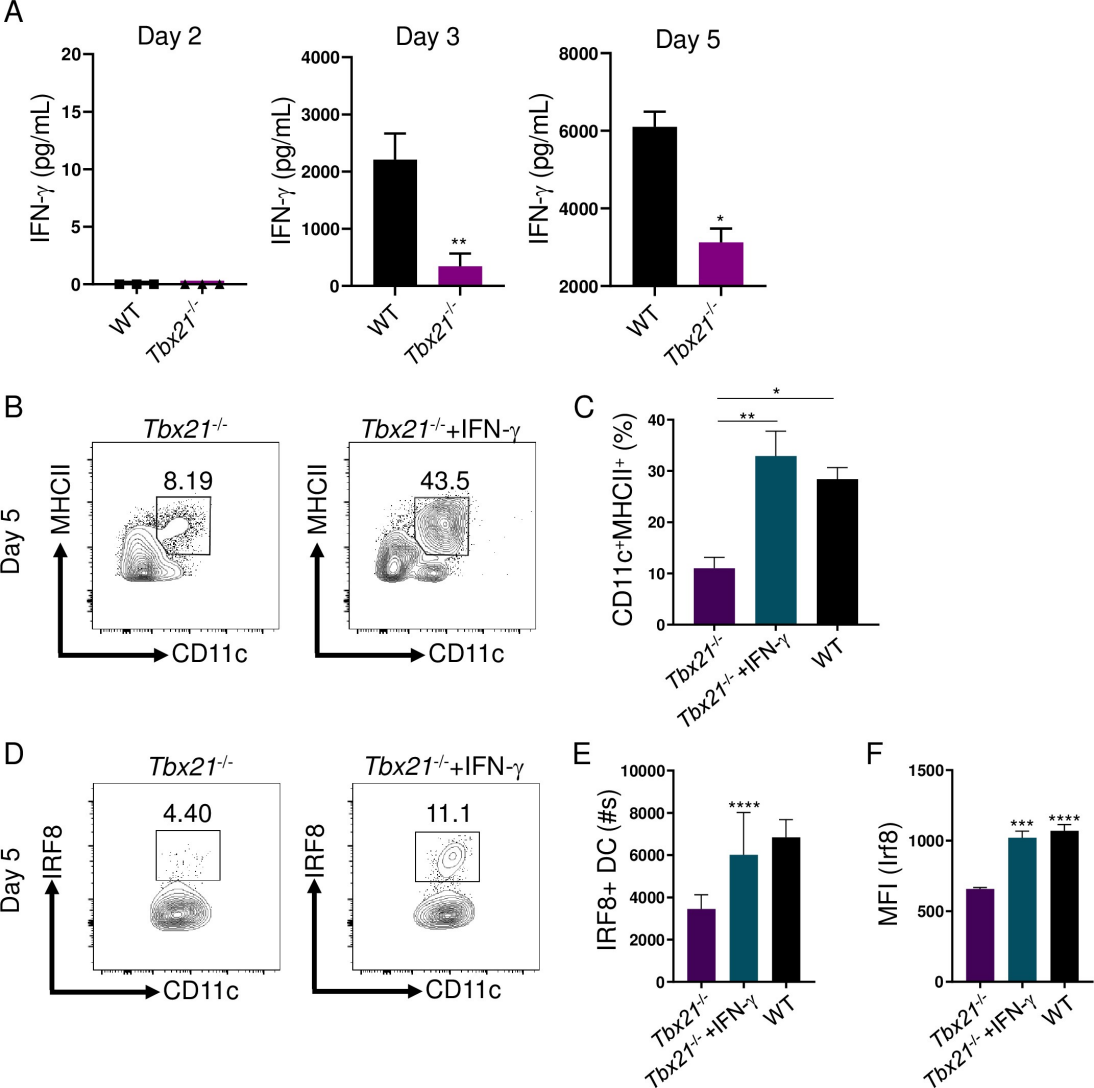
Early T-bet-dependent IFN-γ is critical to maintain inflammatory IRF8+ cDCs1. (**A**) IFN-γ analysis by ELISA of serum in mice following *T*. *gondii* infection on days 2, 3, and 5 post-infection. (**B-F**) *Tbx21*^-/-^ mice were i.p. infected with *T*. *gondii* and treated with or without IFN-γ. (B) Representative contour plots and (C) average frequencies of Lin^-^CD11c^+^MHCII^+^ DCs in the PECs were analyzed on day 5 following infection. (D) Representative contour plots and absolute quantification of (E) Lin^-^CD11c^+^MHCII^+^IRF8^+^ DCs in the PECs were analyzed on day 5 following infection. (F) Mean fluorescent intensity (MFI) of Lin^-^CD11c^+^MHCII^+^ DC IRF8 expression in the PECs was analyzed on day 5 post-infection. Results are representative of three-independent experiments involving at least 3 mice per group. Statistical analyses were done using (A, E, F) unpaired t-test analysis of individual groups or (C) one-way Anova with Tukey’s multiple comparison test, *P<0.05, **P<0.01, ***P<0.001, ****P<0.0001. Error bars, standard error mean.

To identify if the lack of T-bet resulted in impaired early IFN-γ production during *T*. *gondii* infection, we performed a detailed analysis of *T*. *gondii*-mediated IFN-γ production. Our results identified that T-bet was critical for early IFN-γ production on days 3 and 5 of parasite infection (Fig 3A). Therefore, we hypothesized that early IFN-γ is required to maintain DCs during *T*. *gondii* infection. To test our hypothesis, IFN-γ was administered to infected *Tbx21*^-/-^ mice, which not only significantly augmented inflammatory DCs at the site of infection (Fig 3B and 3C), but also rescued IRF8 expression in DCs (Fig 3D–3F). These data demonstrate T-bet-dependent early IFN-γ production is essential for maintaining inflammatory DCs via regulation of IRF8.

### T-bet is essential for ILC1-derived IFN-γ and contributes to NK cell-derived IFN-γ

**Fig 4:**
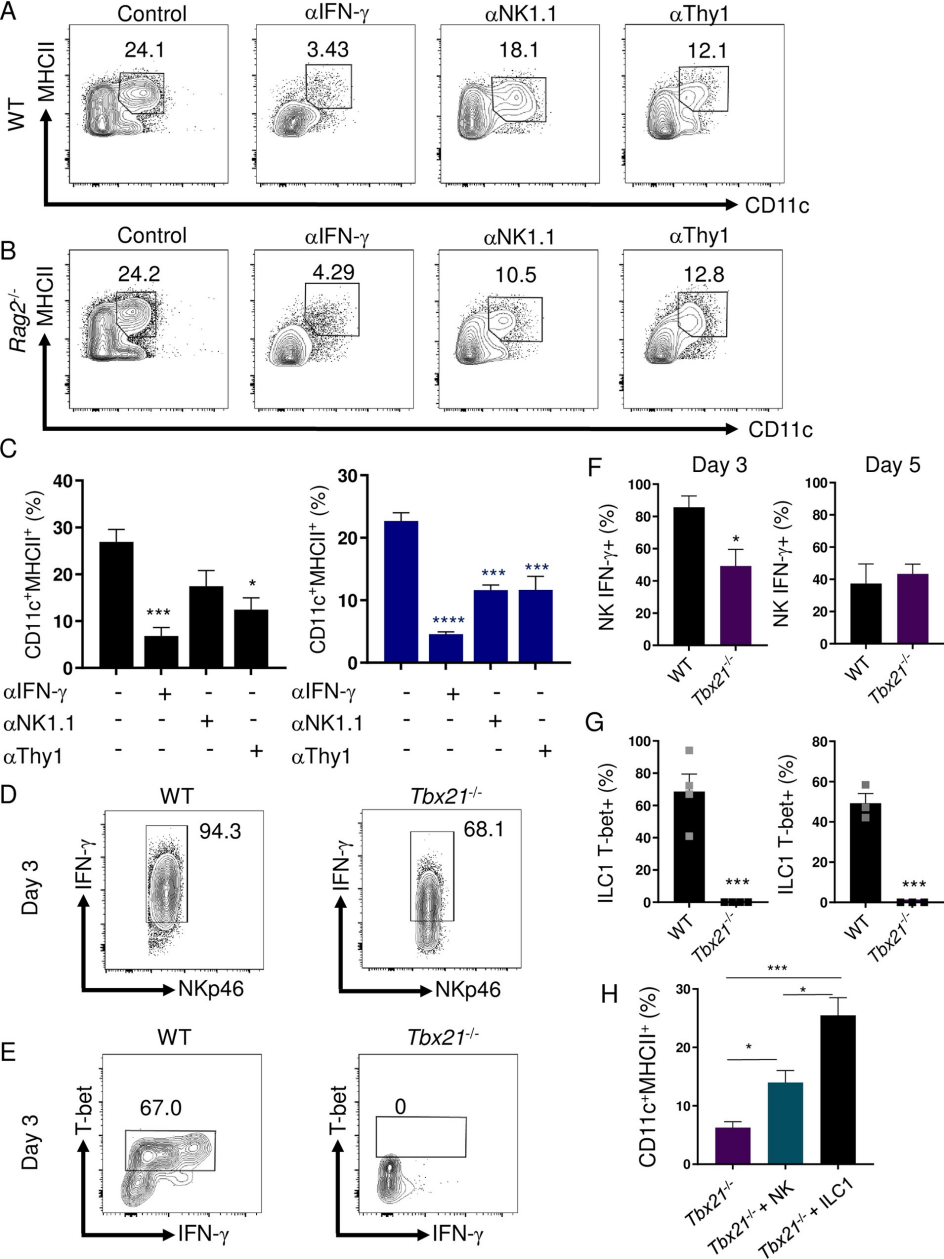
IFN-γ and ILC1s are required for sustaining inflammatory DCs during *T*. *gondii* infection. (**A, B**) WT and *Rag2*^-/-^ mice were i.p. infected with 20 cysts of *T*. *gondii* and then treated with anti-IFN-γ, anti-NK1.1, or anti-Thy1 antibodies during infection. (**C**) Average frequencies of CD11c^+^MHCII^+^ DCs in the PECs were analyzed on day 8 following infection and antibody treatment. (**D-G**) WT and *Tbx21*^-/-^ mice were infected i.p. with 20 cysts of *T*. *gondii*. **(D**) Day 3 representative contour plots and (**F**) average frequencies of CD127-NKp46+IFN-γ+ NK cells in the PECs were analyzed on days 3 and 5 following infection. (E) Day 3 representative contour plots and (**G**) average frequencies of CD127^+^NKp46^+^T-bet^+^ ILC1s cells in the PECs were analyzed on days 3 and 5 following infection. (**H**) Sort-purified NK cells (CD45+CD3-CD19-NKp46+CD127- NKs) and ILC1s (CD45+CD3-CD19-NKp46+CD127+) from the PECs of WT mice (S6C Fig) infected with *T*. *gondii* were adoptively transferred into *Tbx21*^-/-^ mice on day 2 post infection, and the presence of DCs (Lin-CD11c+MHCII+) was analyzed by flow cytometry 3 days later (day 5 post infection). Results are representative of three-independent experiments involving at least 3 mice per group. Statistical analyses were done using unpaired t-test analysis of individual groups, or (C) one-way Anova with Tukey’s multiple comparison test, *P<0.05, ***P<0.001, ****P<0.0001.

To thoroughly define the function of IFN-γ in regulating inflammatory DCs, we examined if neutralizing IFN-γ eliminates their presence at the site of infection. We observed that blocking IFN-γ during *T*. *gondii* infection resulted in a striking reduction of inflammatory DCs in both WT and T cell-deficient mice (Fig 4A–4C). These results revealed that IFN-γ from innate immune cells was sufficient to maintain inflammatory DCs in the absence of T cell-derived IFN-γ. Among innate immune functions, T-bet has been described to play an important role for NK cell function and ILC1 development, and both of these cell types primarily control acute *T*. *gondii* infection via their production of IFN-γ [14–16]. Therefore, we examined IFN-γ production by NKs, ILC1s, neutrophils, T, and B cells on days 3 and 5 of infection and observed that NKs and ILC1s are the primary sources of the effector cytokine during the acute stage of infection (S3A Fig). To characterize the importance of innate IFN-γ for the maintenance of inflammatory DCs, we treated WT and *Rag2*^-/-^ mice with anti-NK1.1 antibodies resulting in a profound loss of inflammatory DCs (Fig 4A–4C), further identifying that innate lymphoid cells are critically involved in sustaining parasite-triggered inflammatory DCs. To further explore if ILC1s were sufficient and necessary for maintaining DCs during infection, we next tested the effects of selective depletion of Thy1+ cells in WT and *Rag2*^-/-^ mice. Depletion of Thy1 expressing cells resulted in a significant decrease of DCs in WT and *Rag2*^-/-^ mice (Fig 4A–4C). Moreover, Thy1-mediated depletion of ILC1s in *Rag2*^-/-^ mice resulted in increased pathogen burdens comparable to levels seen in T-bet-deficient animals (S3B Fig). Our data implicates that either NK cell- or ILC1-derived IFN-γ, in a T-bet-dependent manner, are required to maintain inflammatory DCs during *T*. *gondii* infection.

Our experiments with WT and T-bet-deficient mice revealed that in response to *T*. *gondii* infection, NK cells produced large amounts of IFN-γ in a T-bet-independent manner. This was evident from comparative analysis of IFN-γ production by NK cells in WT and *Tbx21*^-/-^ mice on days 3 and 5 post-infection (Figs 4D, 4F and S4A). Nevertheless, an adoptive transfer of NK cells into T-bet-deficient mice partially rescued the presence of inflammatory DCs (Fig 4H). These results revealed that while T-bet is not absolutely required for IFN-γ production by NK cells during *T*. *gondii* infection, T-bet-regulated production of IFN-γ by NK cells plays a role in the regulation of inflammatory DCs.

Most importantly, we observed robust T-bet expressing ILC1-derived IFN-γ responses during infection in WT mice (Fig 4E and 4G). We identified ILC1s from the peritoneal cavity as CD3^-^CD19^-^Ly6G^-^NKp46^+^CD127^+^ (S6B Fig) and, upon further evaluation, confirmed this population was also NKp46^+^GranzymeB^-^CD49a^+^CD49b^-^CD127^+^CD200R^+^ (S5 Fig), and as anticipated were absent in *Tbx21*^-/-^ mice at all examined time points following *T*. *gondii* infection (Fig 4E and 4G). Therefore, T-bet deficiency resulted in two major defects in innate immunity: a complete lack of ILC1s and inflammatory DCs at the site of infection. We further examined a role for ILC1s in maintenance of inflammatory DCs during *T*. *gondii* infection by analyzing parasite infected lymphocyte-deficient (*Rag2*^-/-^*γc*^-/-^) mice, which also lack ILC1s. We observed that similar to T-bet deficiency, *Rag2*^-/-^*γc*^-/-^ mice had very few inflammatory DCs (S4B Fig), further demonstrating that ILC1s are essential for the presence of inflammatory DCs during parasite infection. Finally, an adoptive transfer of ILC1 into *Tbx21*^-/-^ mice restored inflammatory DCs at the site of infection (Figs 4H and S6C). Taken together, these result suggest that ILC1s play a major role in the regulation of inflammatory DCs.

### IRF8+ DCs are critical for pathogen clearance against intracellular infection

**Fig 5:**
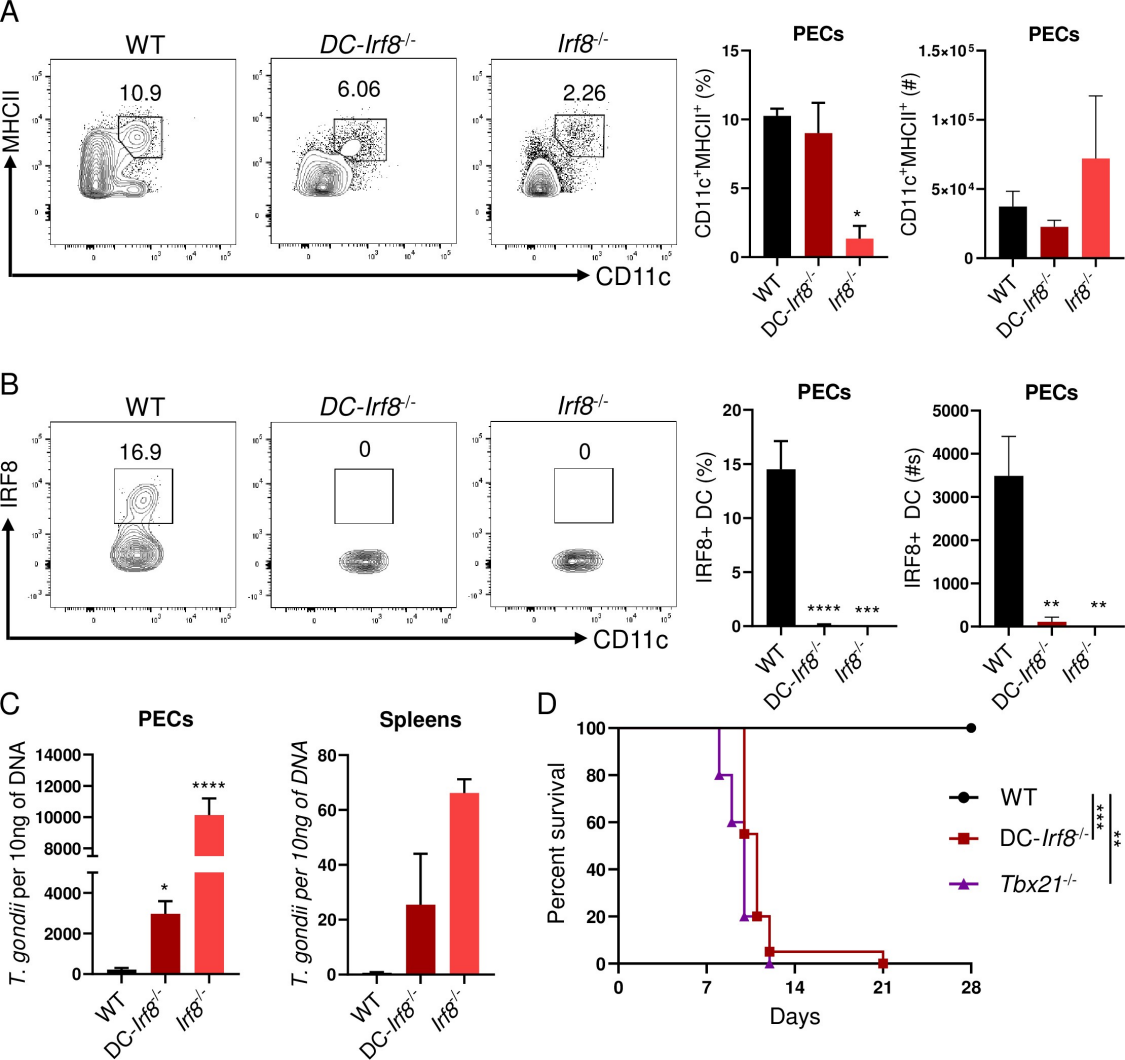
cDC1s are essential for host resistance against intracellular infection. (**A**, **B, C**) WT, DC-*Irf8*^-/-^, and *Irf8*^*-/-*^ mice were infected i.p. with 20 cysts of *T*. *gondii*. (A, B) Representative contour plots, average frequencies, and absolute number of (A) Lin^-^CD11c^+^MHCII^+^ and (B) Lin^-^CD11c^+^MHCII^+^IRF8^+^ DCs in the PECs were analyzed on day 5 following infection. (C) WT, DC-*Irf8*^-/-^, and *Irf8*^-/-^ mice were i.p. infected with *T*. *gondii* and pathogen burden was assessed from PECs and spleen of mice following infection by qPCR on day 5 post-infection. (**D**) Survival of WT (●), *Tbx21*^-/-^ (▲), and DC-*Irf8*^-/-^ (■) mice infected i.p. with 20 cysts of *T*. *gondii*. Results are representative of four independent experiments involving at least 3 mice per group. Statistical analyses were done using (D) Log-rank (Mantel Cox) test or (A-C) one-way Anova with Tukey’s multiple comparison test, *P<0.05, **P<0.01, ***P<0.001, ****P<0.0001. Error bars, standard error mean.

Our data has identified that early ILC1-derived IFN-γ is critical for maintaining IRF8^+^ DCs during infection and limiting parasite replication throughout the host. Therefore, we hypothesized that conditionally deleting IRF8 expression by DCs would result in uncontrolled parasite replication during infection and rapid host mortality. To test our hypothesis, we infected mice with a DC-restricted deficiency of IRF8 by using the CD11c-Cre system (CD11c-Cre x *Irf8*^flox/flox^ mice, DC-*Irf8*^-/-^). Infected DC-*Irf8*^-/-^ mice revealed an overall reduction of total MHCII^+^CD11c^+^ DCs by day 5 post-infection (Fig 5A). Moreover, the absence of IRF8 expression in MHCII^+^CD11c^+^ DCs resulted in dramatically elevated parasite burden both at the site of infection and in the spleen on day 5 post infection (Fig 5B and 5C). We then examined if IRF8-deficient DCs were required for host survival against *T*. *gondii*. Similarly to T-bet-deficient mice, DC-*Irf8*^-/-^ animals succumb to parasite infection rapidly (Fig 5D). Our data establishes that early and rapid T-bet-dependent innate IFN-γ is indispensable for maintaining IRF8^+^ cDC1s, which are required for pathogen clearance and host survival.

## Discussion

The cytokine IFN-γ is critical for triggering cellular anti-parasitic defense mechanisms that are essential for the destruction of the intracellular parasitophorous vacuole and *T*. *gondii* clearance [9,12,36–43]. Classically, the transcription factor T-bet is considered the master regulator for determining the CD4+ T_H_1 lineage and IFN-γ production [7]. However, recent studies revealed that that T-bet is largely dispensable for CD4+ T cell-derived IFN-γ responses [4,5]. Herein we demonstrate that *Tbx21*^-/-^ mice succumb to infection significantly quicker than mice lacking T cells, suggesting an innate T-bet-dependent mechanism of host immunity, critical for survival of the acute stage of infection.

Innate lymphoid cells are critical early responders to intracellular pathogens. During *T*. *gondii* infection, two sets of ILCs are known to have essential roles for host resistance: NK cells and ILC1s. T-bet is associated with the maturation, egress, and effector function of NK cells [15,17,18]. Yet, NK cell development is not impaired in the absence of T-bet in this model [16]. Moreover, *T*. *gondii*-triggered NK cell-derived IFN-γ production plays a critical role for initiating the effector function of myeloid cells that are required for host resistance [13,19,44]. Our results demonstrate that the absence of T-bet does not impede the recruitment of NK cells to the site of infection, and only partially compromised their capability to produce IFN-γ. These data establish that during *T*. *gondii* infection, T-bet plays a limited role for the migration of NK cells to the peritoneum and their IFN-γ production.

Along with NK cells, ILC1s have been shown to be a critical early source of IFN-γ during microbial invasion. Thus, it is imperative to perform clear phenotyping of NK and ILC1 populations. Based on previous studies investigating peritoneal NKs and ILC1s, we defined NKs as NKp46^+^GranzymeB^+^CD49a^-^CD49b^+^CD127^-^CD200R^-^ and ILC1s as NKp46^+^GranzymeB^-^CD49a^+^CD49b^-^CD127^+^CD200R^+^ [22,23]. Early ILC1-derived IFN-γ is critical for host resistance against Mouse Cytomegalovirus (MCMV), *Clostridium*, *Salmonella*, and *T*. *gondii* [14,20–22]. T-bet is required for the maturation and cytokine production of ILC1s [14]. Previous groups have observed that *T*. *gondii* infection mediates ILC1-derived IFN-γ and plays an important role in parasite clearance [14]. By examining *T*. *gondii*-infected *Tbx21*^-/-^ mice, we were able to define a host defense function of T-bet-dependent ILC1s during *T*. *gondii* infection. T-bet-deficiency resulted in the complete loss of ILC1s in comparison to a minor reduction in IFN-γ production by NK cells or CD4+ T cells [5,14]. This suggested that the significant reduction of inflammatory DCs observed in *Tbx21*^-/-^ mice was caused by the absence of T-bet-dependent ILC1-derived IFN-γ. By antibody depletion of ILC1s or IFN-γ neutralization, we revealed that ILC1-derived IFN-γ plays a major role in maintaining inflammatory DCs and restricting parasite growth both locally and in peripheral tissues. Nevertheless, it is important to note that T-bet regulated NK cells can also augment inflammatory DCs at the site of the infection. We also observed that absence of T-bet resulted in the reduction of inflammatory monocytes during the acute stage of the infection (S2B Fig). Thus, the critical function of T-bet-dependent ILC1- and NK cell-derived IFN-γ is to maintain inflammatory DCs at the site of infection.

We and others have previously established that the transcription factor IRF8 is essential for host resistance to *T*. *gondii* [34,45]. IRF8-deficiency resulted in acute susceptibility to *T*. *gondii* due to the absence of cDC1s [46]. Our results revealed that early T-bet-dependent IFN-γ was critical for maintaining inflammatory DCs via regulation of IRF8 expression and DC-specific IRF8 is required for host resistance to the parasite.

This study defines the critical function of early T-bet-dependent IFN-γ in sustaining inflammatory IRF8^+^ DCs during intracellular pathogen infection. Our results demonstrate continuous crosstalk between DCs and ILC1s mediated by IL-12 and IFN-γ, where DC-derived IL-12 triggers an early IFN-γ response from ILC1s and NK cells and IFN-γ produced by innate lymphoid cells is essential for maintaining inflammatory DCs during infection via regulation of IRF8.

## Material and methods

### Ethics statement

All mice were maintained at in the pathogen-free American Association of Laboratory Animal Care-accredited animal facility at the University of Rochester Medical Center, Rochester, NY.

All animal experimentation (animal protocol #102122) has been reviewed and approved by the University Committee on Animal Resources (UCAR), the Institutional Animal Care and Use Committee (IACUC).

### Animals

C57BL/6, *Rag2*^-/-^, *Tbx21*^-/-^, CD11c-Cre, *Irf8*^flox/flox^, and *Tbx21*^flox/flox^ mice were obtained from Jackson Laboratory (Bar Harbor, ME) and *Rag2*^-/-^*γc*^-/-^ mice were obtained from Taconic (Rensselaer, NY). All control and experimental mice were age- and sex-matched within all individual experiments. This study included both male and female mice, and the data derived from male and female mice identified no sex-specific differences in the performed experiments.

#### *Toxoplasma gondii* infection and qPCR

All mice were i.p. infected with an average of 20 *T*. *gondii* cysts of the ME49 strain. At days 0, 3, 5, and 8 post-infection, the animals were necropsied. In some experiments, mice were injected i.p. with 50 ng of IFN-γ (R&D Systems) on days 0, 1, 2, and 3. In some experiments, mice were injected i.p. with 200 μg of anti-IFN-γ (BioxCell) 500 μg of anti-NK1.1 (BioxCell), or 200 μg of anti-Thy1.2 (BioxCell) on days 0, 3, 5. To determine *T*. *gondii* pathogen loads, total genomic DNA from animal tissue was isolated by using the DNeasy Blood and Tissue Kit (Qiagen) according to manufacturer’s instructions. PCR were performed by using SSOFast Eva Green Supermix (BioRad). Samples were measured by qPCR using a MyiQ Real-Time PCR Detection System (BioRad), and data from genomic DNA was compared with a defined copy number standard of the *T*. *gondii* gene *B1*.

#### ELISA analysis

The IFN-γ and IL-12/23p40 concentration in the sera or peritoneal exudate fluid (PEF) was analyzed by standard sandwich ELISA kit according to manufacturer’s instructions (ThermoFisher).

#### Measurements of immune cells responses

To assay the responses of mice infected with *T*. *gondii*, the PECs were harvested from C57BL/6, *Rag2*^-/-^, *Tbx21*^-/-^, DC-*Irf8*^-/-^, DC-*Tbx21*^-/-^, and *Rag2*^-/-^*γc*^-/-^ mice on days 0, 3, 5, or 8 post-infection. To examine neutrophils, T cells, B cells, NK cells and ILC1s responses, single-cell suspension of PECs were restimulated with PMA (20 ng/mL) and ionomycin (1 μg/mL) (Sigma-Aldrich) for 4 hours in the presence of GolgiPlug (Brefeldin A, BD Biosciences). After isolation and *in vitro* restimulation, the cells were washed once in phosphate-buffered saline and stained with Zombie Yellow (BioLegend) to assess live vs. dead status of cells. Cells were then washed with phosphate-buffered saline + 1% fetal bovine serum and stained with fluorochrome-conjugated antibodies. For intracellular staining and subsequent washing, cells were permeabilized overnight at 4°C with the Foxp3/Transcription Factor Staining Buffer Set according to the manufacturer’s instructions (ThermoFisher).

For the adoptive transfer experiments, sort-purified (50,000 per recipient) NK cells (CD45^+^CD3^-^CD19^-^NKp46^+^CD127^-^) and ILC1 (CD45^+^CD3^-^CD19^-^NKp46^+^CD127^+^) were prepared from the PECs of *T*. *gondii* infected WT mice and adoptively transferred into *Tbx21*^-/-^ mice infected with the parasite two days earlier.

Cells were sorted using a FACSAria II cell sorter (BD Biosciences). Cell fluorescence was measured using an LSRII flow cytometer (BD Biosciences), and data were analyzed using FlowJo Software (Tree Star, Ashland, OR).

### Statistical analysis

All data were analyzed with Prism (Version 8; GraphPad, La Jolla, CA). These data were considered statistically significant when *P*-values were <0.05.

## Supporting information

S1 Fig T-bet is required for maintaining inflammatory DCs during *T*. *gondii* infections. (**A-D**) WT, *Rag2*^-/-^, and *Tbx21*^-/-^ mice were infected i.p. with 20 cysts of *T*. *gondii*. (A) Representative contour plots of Lin-CD11c+MHCII+ DCs and their average frequencies from WT and *Tbx21*^-/-^ PECs that were harvested on day 3 post-infection. (B) Representative contour plots of Lin-CD11c+MHCII+ and (D) IRF8+ DCs from *Rag2*^-/-^ PECs that were harvested on days 0, 5, and 8. (C) Absolute number of Lin-CD11c+MHCII+ and (E) IRF8+ DCs in the PECs were analyzed on days 0, 5, and 8 following infection. Results are representative of three-independent experiments involving at least 3 mice per group. Statistical analyses were done using one-way Anova with Tukey’s multiple comparison test, *P<0.05, **P<0.01, ***P<0.001, ****P<0.0001. Error bars, standard error mean.
(TIF)

S2 Fig Intrinsic T-bet expression is not required for MHCII+CD11c+ DCs. (**A**) DC-*Tbx21*^-/-^ mice were infected i.p. with 20 cysts of *T*. *gondii*. (A) Representative contour plots of Lin-CD11c+MHCII+ and IRF8+ DCs from DC-*Tbx21*^-/-^ PECs that were harvested on day 5 post-infection. (**B**-**C**) WT and *Tbx21*^-/-^ mice were i.p. infected with *T*. *gondii*. (B) Frequency and absolute number of CD115+Ly6C^Hi^ monocytes from WT and *Tbx21*^-/-^ mice in the PECs were analyzed on days 0, 3, and 5 following infection by flow cytometry. (C) IL-12/23p40 analysis by ELISA of serum and PEF in mice following *T*. *gondii* infection on day 5 post-infection. Statistical analyses were done using unpaired t-test analysis of individual groups, *P<0.05, **P<0.01, ***P<0.001, ****P<0.0001. Error bars, standard error mean.
(TIF)

S3 Fig ILC1s and NK cells are critical innate sources for IFN-γ during *T*. *gondii* infection. (**A**) WT mice were infected i.p. with 20 cysts of *T*. *gondii*. (A) Frequency of IFN-γ expressing CD19+ B cells, Ly6G+ neutrophils, CD3+ T cells, NKp46+CD127- NKs, and NKp46+CD127+ ILC1s from the PECs of WT mice analyzed on days 3 and 5 following infection by flow cytometry. (**B**) Parasite burden was assessed from PECs and spleen of *Rag2*^-/-^ mice treated with or without anti-Thy1 antibody by qPCR. Results are representative of three-independent experiments involving at least 3 mice per group. Statistical analyses were done using unpaired t-test analysis of individual groups, *P<0.05, **P<0.01. Error bars, standard error mean.
(TIF)

S4 Fig T-bet-dependent and -independent NK-derived IFN-γ responses during *T*. *gondii* infection. (**A**, **B**) WT and *Tbx21*^-/-^ mice were infected i.p. with 20 cysts of *T*. *gondii*. Average frequencies of (A, B) CD127-NKp46+IFN-γ+ NK cells in the PECs were analyzed on days 3 and 5 following infection. (**C**) Representative contour plots of Lin-CD11c+MHCII+ DCs from *Rag2*^-/-^*γc*^-/-^ PECs harvested on days 0 and 5 post-i.p. *T*. *gondii* infection. (**D**) Sort-purified NK cells (CD45+CD3-CD19-NKp46+CD127- NKs) and ILC1s (CD45+CD3-CD19-NKp46+CD127+) from the PECs of WT mice infected with *T*. *gondii* were adoptively transferred into *Tbx21*^-/-^ mice on day 2 post infection, and the presence of DCs (Lin-CD11c+MHCII+) was analyzed by flow cytometry 3 days later (day 5 post infection). Results are representative of three-independent experiments involving at least 3 mice per group. Statistical analyses were done using unpaired t-test analysis of individual groups, *P<0.05, ***P<0.001. Error bars, standard error mean.
(TIF)

S5 Fig ILC1s are absent from *Tbx21*^-/-^ mice during *T*. *gondii* infection. (**A**-**C**) WT and *Tbx21*^-/-^ mice were infected i.p. with 20 cysts of *T*. *gondii* and PECs were assessed for ILC1s. (A, B) Average frequencies and absolute number of CD45^+^CD3^-^CD19^-^Ly6G^-^NKp46^+^GranzymeB^-^CD49b^-^CD49a^+^CD127^+^CD200R^+^ in the PECs were analyzed on day 5 following infection. (C) Representative gating strategy for ILC1s. Statistical analyses were done using unpaired t-test analysis of individual groups, **P<0.01. Error bars, standard error mean.
(TIF)

S6 Fig Representative gating strategies for DC and ILC1. (A) DCs in the peritoneal cavity were defined as lineage-negative (CD3^-^CD19^-^NK1.1^-^; Lin^-^) CD11c^+^MHCII^+^ cells. (B) The peritoneal cavity ILC1s were defined as CD45+CD3-CD19-Ly6G-NKp46+CD127+. (C) Post sort analysis of the peritoneal NK cells (left) and ILC1 (right).
(TIF)
