# Supplementary figures and images for: T-bet-dependent ILC1- and NK cell-derived IFN-γ mediates cDC1-dependent host resistance against Toxoplasma gondii
Source: PLoS Pathog. 2021 Jan 19;17(1):e1008299. doi: 10.1371/journal.ppat.1008299 (PMC7875365; doi:10.1371/journal.ppat.1008299)

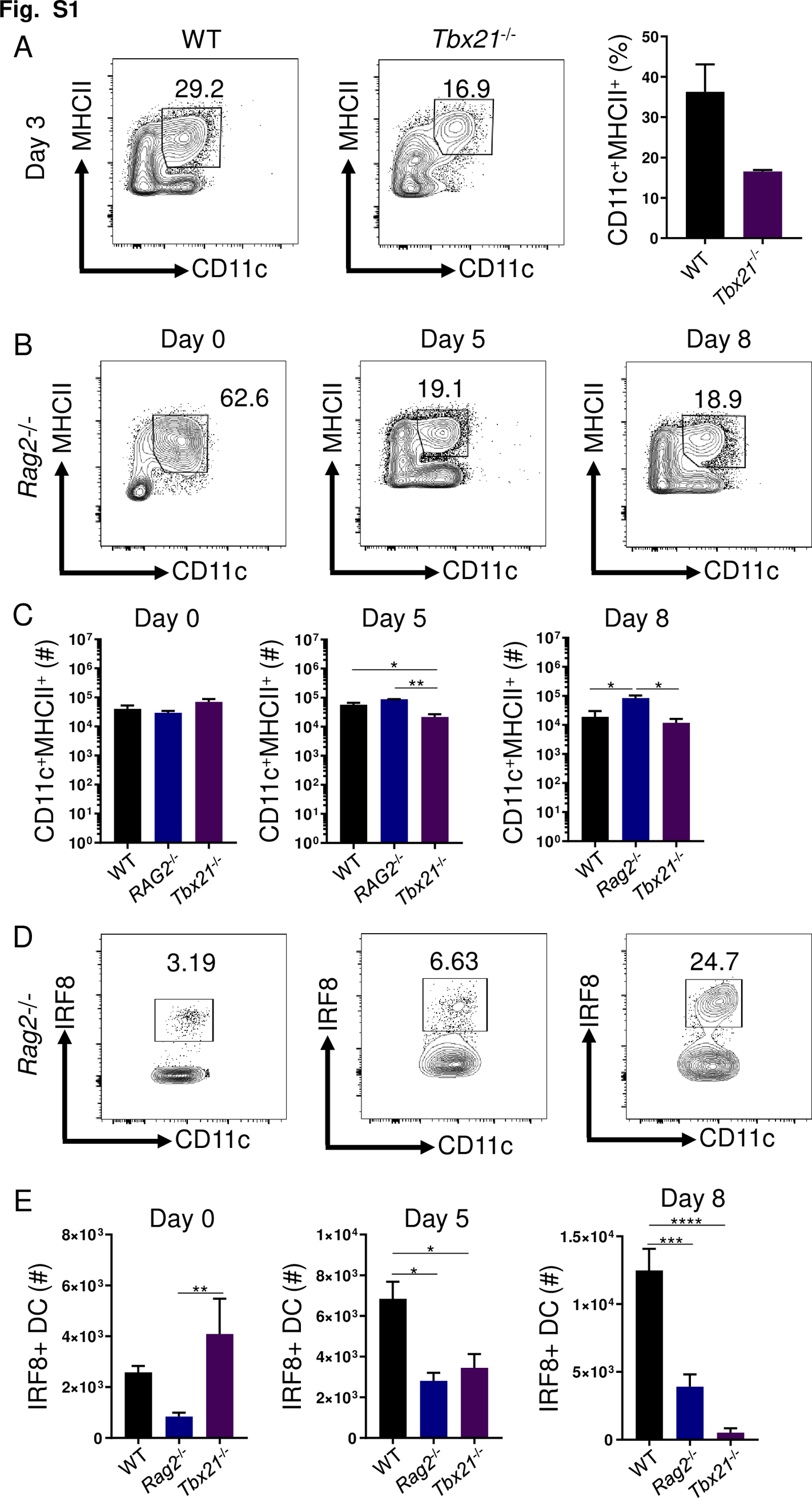

Supplement: S1 Fig — (A-D) WT, Rag2-/-, and Tbx21-/- mice were infected i.p. with 20 cysts of T. gondii. (A) Representative contour plots of Lin-CD11c+MHCII+ DCs and their average frequencies from WT and Tbx21-/- PECs that were harvested on day 3 post-infection. (B) Representative contour plots of Lin-CD11c+MHCII+ and (D) IRF8+ DCs from Rag2-/- PECs that were harvested on days 0, 5, and 8. (C) Absolute number of Lin-CD11c+MHCII+ and (E) IRF8+ DCs in the PECs were analyzed on days 0, 5, and 8 following infection. Results are representative of three-independent experiments involving at least 3 mice per group. Statistical analyses were done using one-way Anova with Tukey’s multiple comparison test, *P<0.05, **P<0.01, ***P<0.001, ****P<0.0001. Error bars, standard error mean. (TIF) [file ppat.1008299.s001.tif]

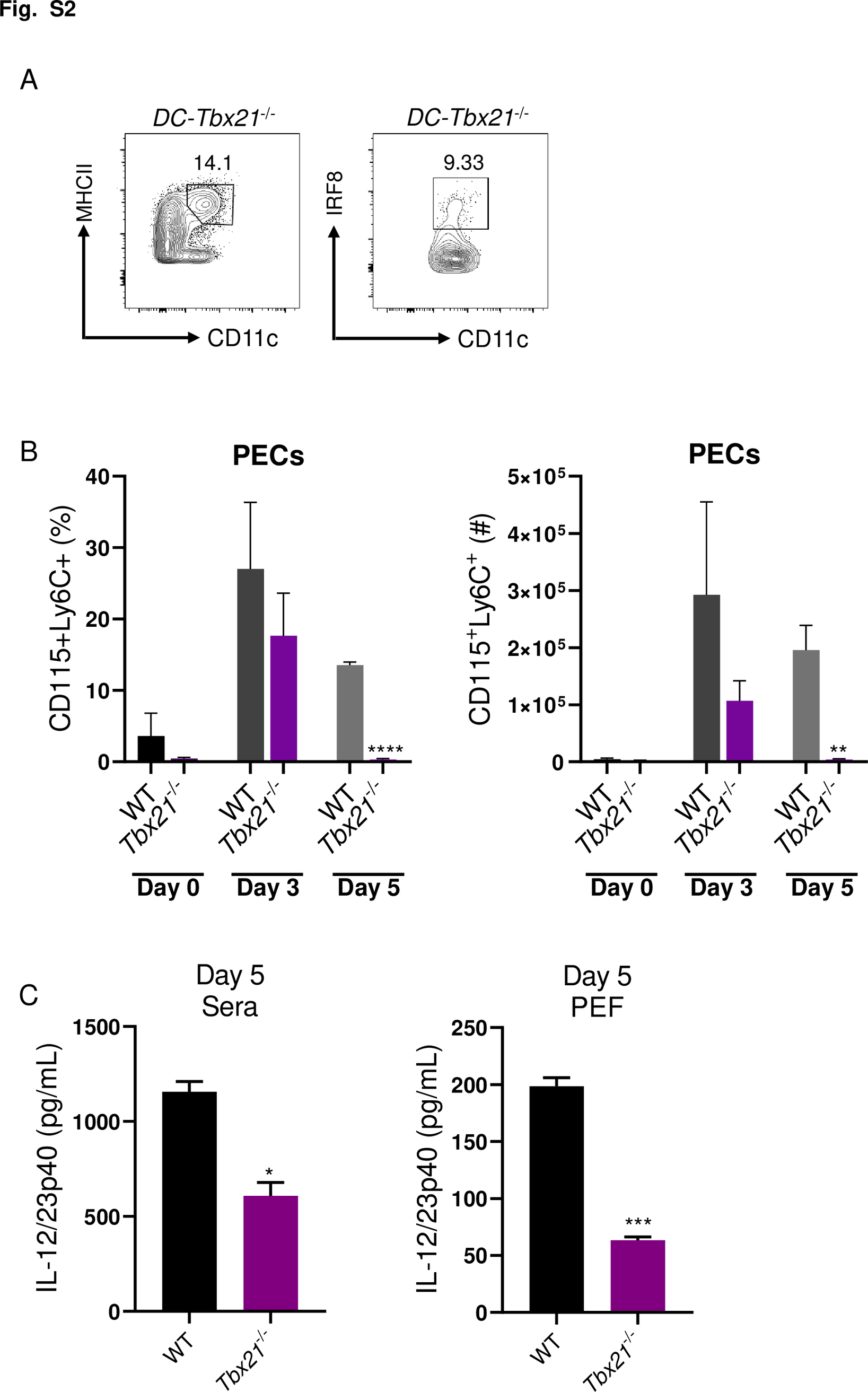

Supplement: S2 Fig — (A) DC-Tbx21-/- mice were infected i.p. with 20 cysts of T. gondii. (A) Representative contour plots of Lin-CD11c+MHCII+ and IRF8+ DCs from DC-Tbx21-/- PECs that were harvested on day 5 post-infection. (B-C) WT and Tbx21-/- mice were i.p. infected with T. gondii. (B) Frequency and absolute number of CD115+Ly6CHi monocytes from WT and Tbx21-/- mice in the PECs were analyzed on days 0, 3, and 5 following infection by flow cytometry. (C) IL-12/23p40 analysis by ELISA of serum and PEF in mice following T. gondii infection on day 5 post-infection. Statistical analyses were done using unpaired t-test analysis of individual groups, *P<0.05, **P<0.01, ***P<0.001, ****P<0.0001. Error bars, standard error mean. (TIF) [file ppat.1008299.s002.tif]

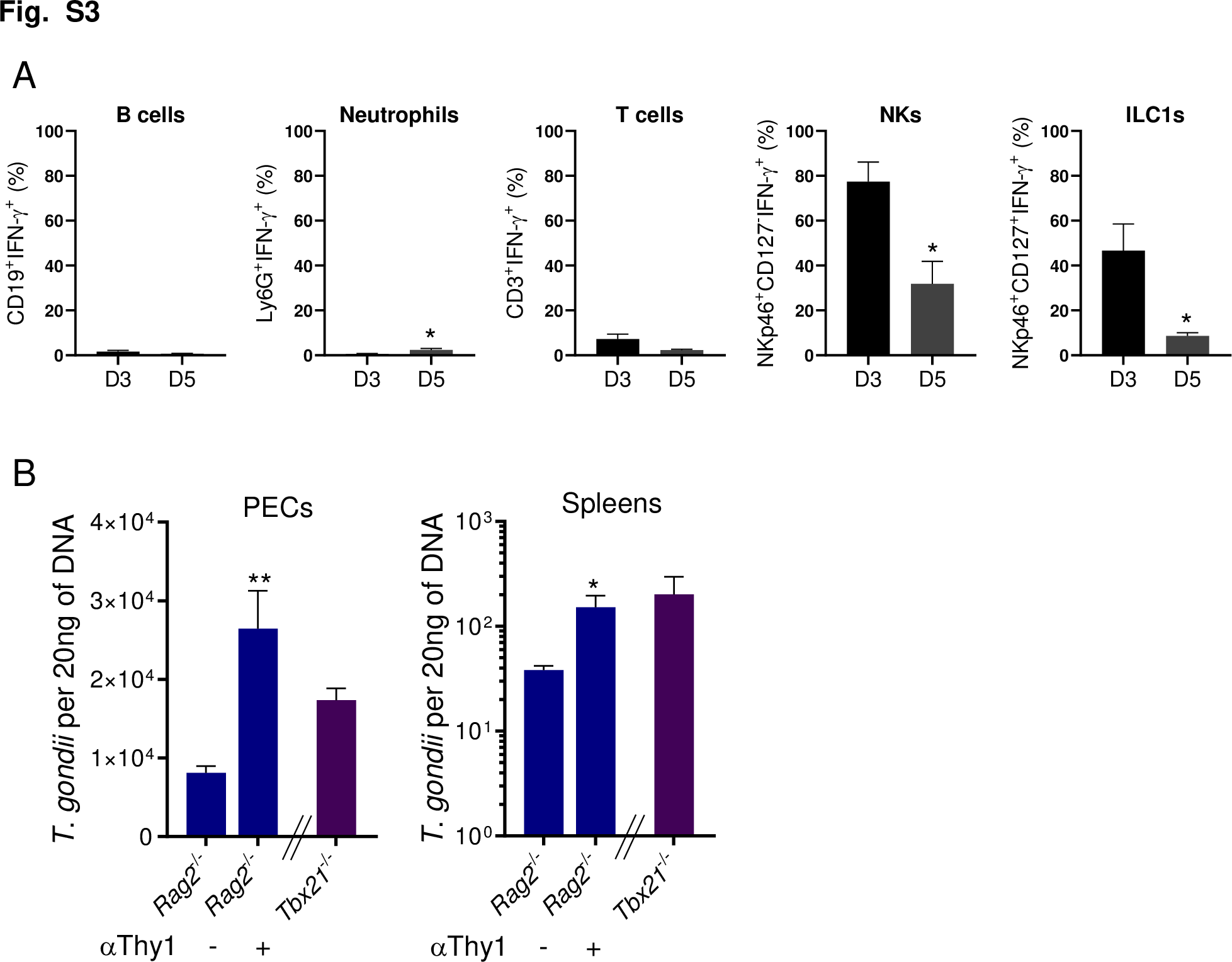

Supplement: S3 Fig — (A) WT mice were infected i.p. with 20 cysts of T. gondii. (A) Frequency of IFN-γ expressing CD19+ B cells, Ly6G+ neutrophils, CD3+ T cells, NKp46+CD127- NKs, and NKp46+CD127+ ILC1s from the PECs of WT mice analyzed on days 3 and 5 following infection by flow cytometry. (B) Parasite burden was assessed from PECs and spleen of Rag2-/- mice treated with or without anti-Thy1 antibody by qPCR. Results are representative of three-independent experiments involving at least 3 mice per group. Statistical analyses were done using unpaired t-test analysis of individual groups, *P<0.05, **P<0.01. Error bars, standard error mean. (TIF) [file ppat.1008299.s003.tif]

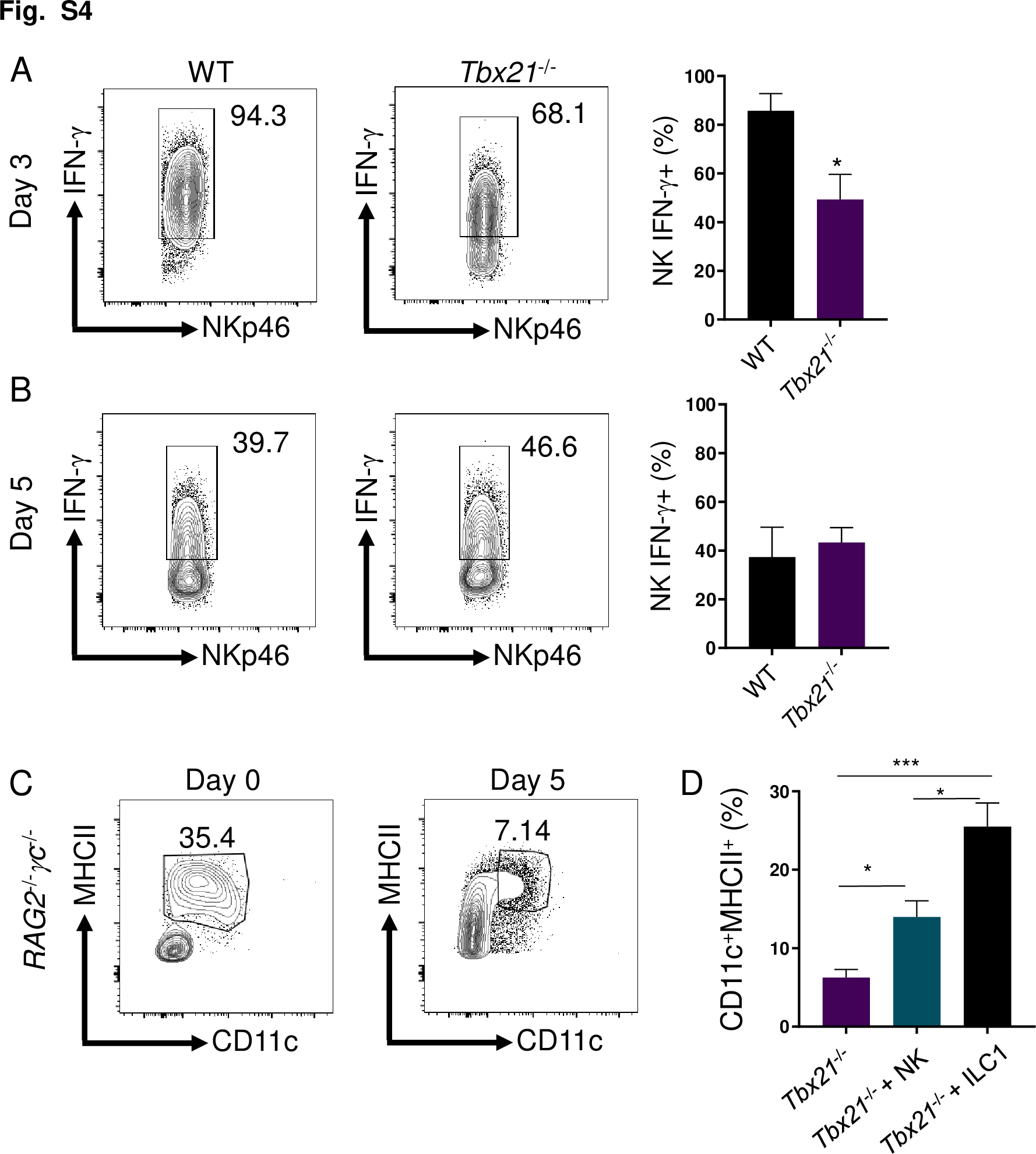

Supplement: S4 Fig — (A, B) WT and Tbx21-/- mice were infected i.p. with 20 cysts of T. gondii. Average frequencies of (A, B) CD127-NKp46+IFN-γ+ NK cells in the PECs were analyzed on days 3 and 5 following infection. (C) Representative contour plots of Lin-CD11c+MHCII+ DCs from Rag2-/-γc-/- PECs harvested on days 0 and 5 post-i.p. T. gondii infection. (D) Sort-purified NK cells (CD45+CD3-CD19-NKp46+CD127- NKs) and ILC1s (CD45+CD3-CD19-NKp46+CD127+) from the PECs of WT mice infected with T. gondii were adoptively transferred into Tbx21-/- mice on day 2 post infection, and the presence of DCs (Lin-CD11c+MHCII+) was analyzed by flow cytometry 3 days later (day 5 post infection). Results are representative of three-independent experiments involving at least 3 mice per group. Statistical analyses were done using unpaired t-test analysis of individual groups, *P<0.05, ***P<0.001. Error bars, standard error mean. (TIF) [file ppat.1008299.s004.tif]

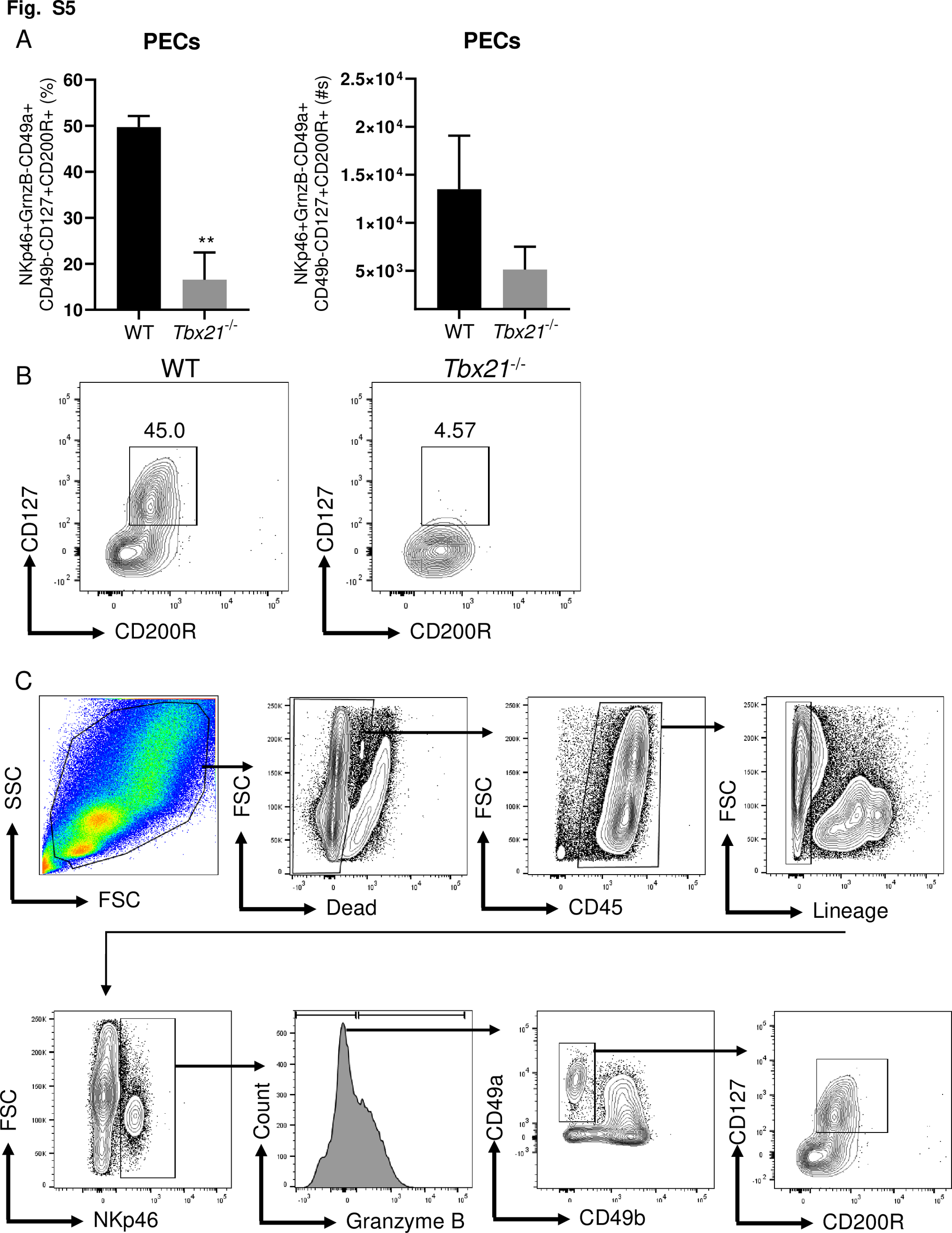

Supplement: S5 Fig — (A-C) WT and Tbx21-/- mice were infected i.p. with 20 cysts of T. gondii and PECs were assessed for ILC1s. (A, B) Average frequencies and absolute number of CD45+CD3-CD19-Ly6G-NKp46+GranzymeB-CD49b-CD49a+CD127+CD200R+ in the PECs were analyzed on day 5 following infection. (C) Representative gating strategy for ILC1s. Statistical analyses were done using unpaired t-test analysis of individual groups, **P<0.01. Error bars, standard error mean. (TIF) [file ppat.1008299.s005.tif]

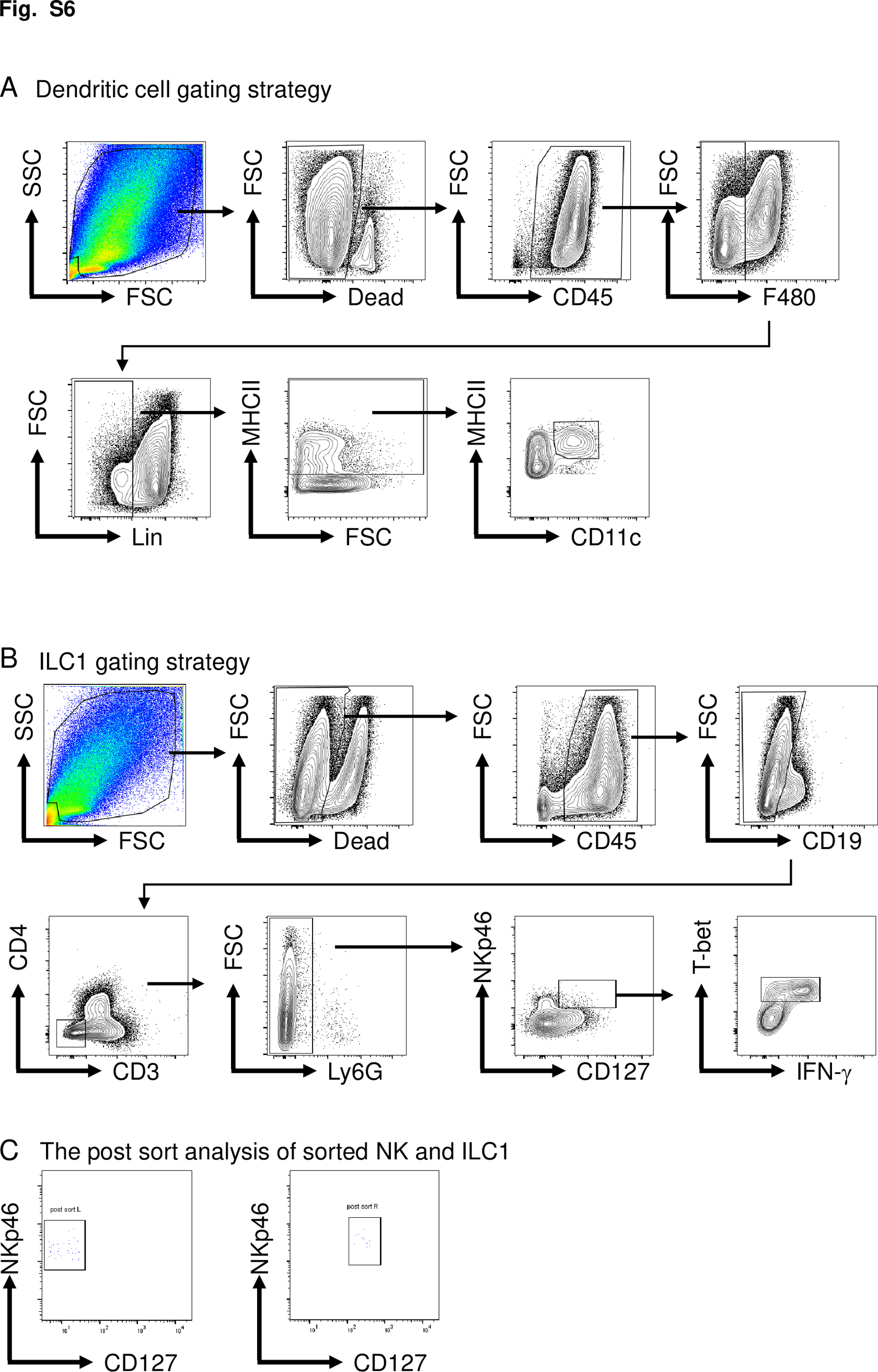

Supplement: S6 Fig — (A) DCs in the peritoneal cavity were defined as lineage-negative (CD3-CD19-NK1.1-; Lin-) CD11c+MHCII+ cells. (B) The peritoneal cavity ILC1s were defined as CD45+CD3-CD19-Ly6G-NKp46+CD127+. (C) Post sort analysis of the peritoneal NK cells (left) and ILC1 (right). (TIF) [file ppat.1008299.s006.tif]
